# The Climates of the United States in Relation to Tuberculosis

**Published:** 1874-01-15

**Authors:** 


The Climates of the United States in Relation to Tuberculosis.—Dr Charles Denison, author of the very interesting communication, relative to the climate of Colorado, which we publish in this number of The Examiner, has undertaken a very important and much needed work.

Being obliged, on account of ill-health, to remove from his home in the East to Denver, he has entered upon a thorough study of the climates of the various parts of the United States, especially in their relation to phthisis—causative, curative, or palliative. In the carrying out of this work, he is endeavoring to place himself in communication with leading physicians in all parts of the country, by means of a circular-letter, containing a series of carefully-arranged questions.

It is to be hoped that physicians generally will respond promptly and fully to the inquiries, as the replies, when gathered together, will undoubtedly bring forward facts of the greatest value and importance relative to the effects of climate on disease.

The Doctor we know to be well fitted for the task he has undertaken, both by natural ability and education. Unbiased and free from prejudice, he is not working to bring into prominence, or to present the claims, of some particular locality as a health-resort, but is honestly endeavoring to carry out the work thoroughly and systematically.

Dr. Denison is especially desirous of corresponding with such physicians as are more particularly interested in the subject of climatic influences, in order to avail himself of their advice and assistance. F. H. D.

---

Means for the Removal of Syphilitic Pigment Stains.—M. Langlebert suggests (*Lyon Medical*, from *Gazette des Hopitaux*) the application of blisters to old stains of syphilitic origin, and the continuance of the suppuration, by the subsequent use of stimulating dressings, for a week. In this way he has caused brown stains, of several years' duration, to disappear.—*Boston Med. and Surg. Jour.*

